# *Cirsium arvense* management with electrical weed control and clopyralid

**DOI:** 10.1002/ps.70736

**Published:** 2026-04-18

**Authors:** Luisa Carolina Baccin, Marcelo L. Moretti

**Affiliations:** ^1^ Department of Horticulture Oregon State University Corvallis OR USA

**Keywords:** electrophysical weed control, integrated weed management, nonchemical weed control, perennial weed

## Abstract

**BACKGROUND:**

Canada thistle [*Cirsium arvense* (L.) Scop.] is a perennial weed that is difficult to control due to its extensive root system and prolific seed production. Although herbicides such as clopyralid provide effective control, they are not suitable for all production systems. Electrical weed control (EWC) is a nonchemical method that applies high voltage to plant tissues, causing irreversible cellular damage through loss of cellular integrity. This study evaluated the efficacy of EWC using a commercial tractor‐mounted unit (EH30; Zasso), clopyralid, and integrated EWC–clopyralid programs for managing *C. arvense*, including single and sequential applications of each approach.

**RESULTS:**

In Corvallis, EWC provided 39% and 53% control with one and two applications, respectively, whereas in Canby, control increased from 38% with a single application to 94% with two applications. Clopyralid provided 96% control with a single application and 95% with two applications in Corvallis, compared with 43% and 78% in Canby. Two EWC applications reduced shoot biomass by 95% in Canby. Combined treatments were highly effective: EWC followed by clopyralid achieved 89% control in Corvallis and 87% in Canby, and the reverse sequence achieved 98% control.

**CONCLUSION:**

EWC provided transient control of *C. arvense*, and integration with clopyralid improved control, supporting its role in integrated weed management. Because *C. arvense* is a perennial species, multiyear studies are needed to determine the long‐term effectiveness of EWC. © 2026 The Author(s). *Pest Management Science* published by John Wiley & Sons Ltd on behalf of Society of Chemical Industry.

## Graphical abstract

Electrical weed control (EWC) treatments reduced *Cirsium arvense* biomass by ≤95% and suppressed new shoot emergence by 70–95%, achieving control comparable to clopyralid. Sequential or integrated applications enhanced efficacy, demonstrating EWC's potential as a nonchemical tool for perennial weed management in orchard systems.

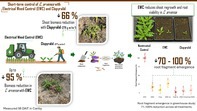

## 1 INTRODUCTION

Canada thistle [*Cirsium arvense* (L.) Scop.] is a perennial weed that poses significant challenges in perennial cropping systems.^1^ The plant exhibits aggressive growth, with deep and persistent root systems reaching ≤3 m in depth. The propagative roots produce adventitious root buds, allowing new shoots to emerge continuously. It reproduces and disperses through prolific seed production, with seeds remaining viable in the soil for ≤22 years and contributing to a long‐lasting seed bank.^2^ Owing to these characteristics, *C. arvense* reduces crop yields and increases management costs in both conventional and organic systems. Yield losses can be substantial and vary with crop and infestation level; in winter wheat, dense *C. arvense* patches have reduced grain yield to 28–71%.^3^ Interest in nonchemical weed management has grown over the years, reflecting efforts to reduce herbicide reliance and adopt more sustainable practices.^4^

In conventional systems, clopyralid, a Weed Science Society of America (WSSA) Group 4 auxinic herbicide, provides selective postemergence control of broadleaf weeds, particularly several Asteraceae species (e.g. *Cirsium* spp., *Ambrosia* spp.), Polygonaceae (e.g. *Rumex* spp. and *Persicaria* spp.), Solanaceae (e.g. *Solanum* spp.) and some Fabaceae (e.g. *Trifolium* spp.) species depending on use pattern and rate.^5^, ^6^, ^7^ However, in hazelnut (*Corylus avellana* L.), clopyralid use is restricted to nonbearing crops and a maximum annual use rate of 0.56 kg a.i. ha^−1^.^8^ Clopyralid also is labeled for use in several other perennial crops (e.g. pastures, and tree fruits), but similar application constraints limit its long‐term effectiveness. Additionally, although resistance to clopyralid is relatively uncommon, four cases have been documented globally, including resistant biotypes of *Centaurea stoebe* (L.) in Canada, *Soliva sessilis* (Ruiz & Pav.) and *Chenopodium album* (L.) in New Zealand, and *Ambrosia artemisiifolia* (L.) in Michigan, USA,^9^ highlighting the potential risks associated with overreliance on this herbicide. Beyond these reports, auxinic herbicide resistance also has been documented in *C. arvense*, with resistant populations confirmed in Hungary (MCPA and 2,4‐D) and Sweden (MCPA), further underscoring the need for diversified management approaches.^9^

Weed management in organic production systems currently relies on mechanical cultivation, cover cropping and organic contact herbicides. These strategies are poorly suited for *C. arvense* control as the species spread through a persistent creeping root system, and cultivation frequently accelerates its spread by stimulating regrowth by fragmenting and distributing viable root pieces.^6^ Furthermore, herbicides currently approved for use in organic production are strictly contact materials that do not translocate to underground structures, limiting their efficacy. Thus, organic growers in the Pacific Northwest listed *C. arvense* as one of the most problematic weeds.^10^, ^11^, ^12^ These limitations have driven interest in integrated weed management (IWM), a method that combines different tactics (cultural, mechanical, biological and when allowed, chemical)^13^, ^14^ to impose repeated stress on belowground reserves of *C. arvense*. Organic IWM programs typically aim to repeatedly deplete belowground reserves through intensive mechanical disturbance and increased crop competition via suppressive covers and crop rotation, including perennial pasture and forage crops^15^, ^16^, ^17^ that can reduce stem density and biomass over time. Biological control also has received attention in organic systems, including evaluation of fungal agents (e.g. *Puccinia punctiformis* F. Strauss Röhl),^18^, ^19^ but reliable suppression and implementation constraints remain barriers to stand‐alone control in agricultural areas. Collectively, available evidence indicates that single tactics rarely provide durable control, whereas integrated programs that combine competition with repeated, well‐timed physical stress are more likely to achieve higher control.^6^ Consequently, there is a need for innovative, nonchemical alternatives that can effectively target both above‐ and belowground structures of *C. arvense*.

Electrical weed control (EWC) is an electrophysical weed management technology that applies high‐voltage electric current to weed foliage.^20^ In this system, one electrode contacts the target plant, allowing the current to pass through its tissues into the soil, while a second electrode completes the circuit by contacting the soil or nearby plants.^21^ Recent field evaluations have demonstrated that commercial EWC systems can achieve weed biomass reductions comparable to herbicides in perennial cropping systems.^22^

**Figure 1:**
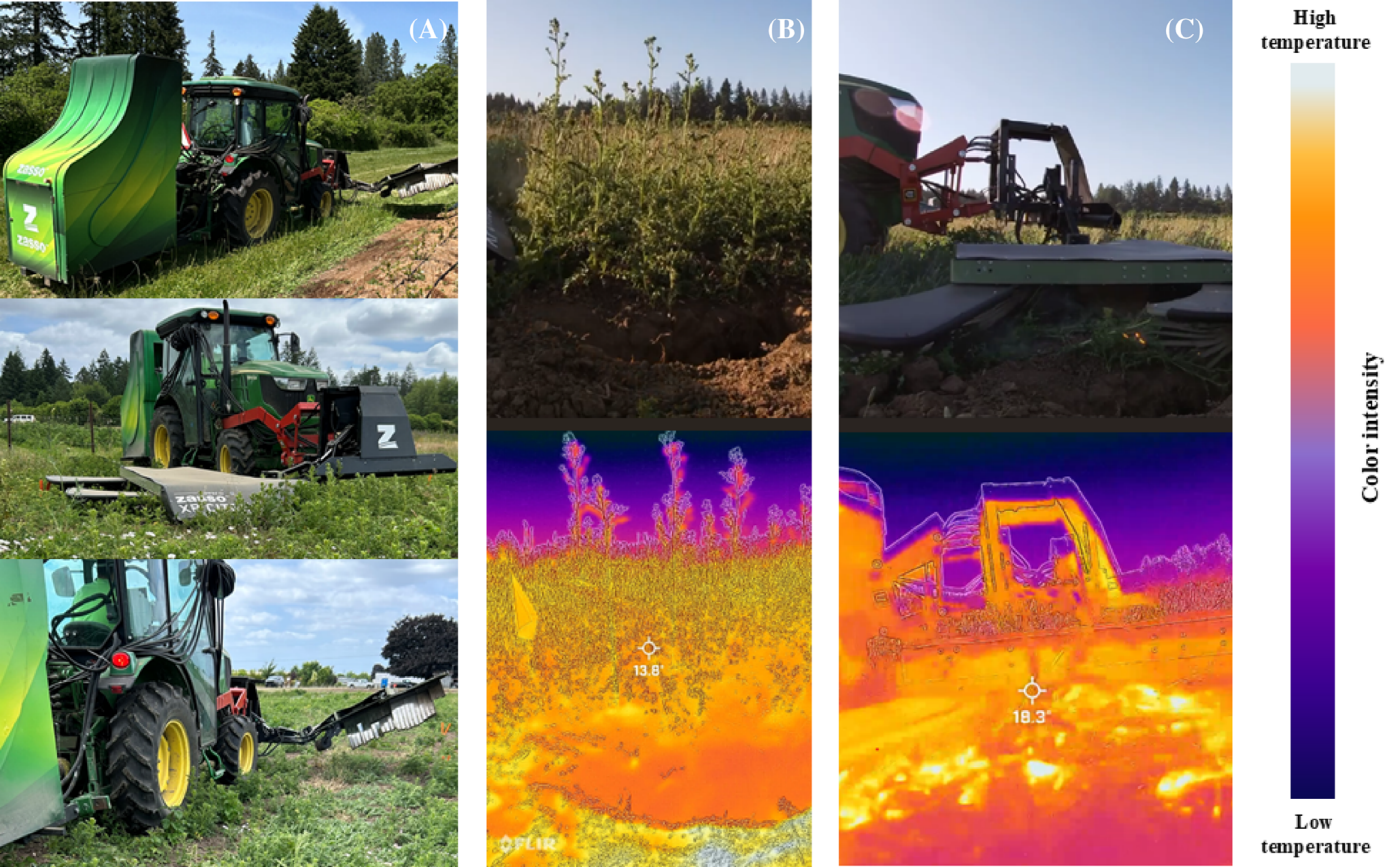
Illustrative images of the EWC system and thermal responses during operation. (A) Electrical weeder EH‐30 Thor (Zasso), mounted on a 77 kW tractor (5100GN; John Deere), with a 1.2‐m applicator attached at the front. (B) and (C) Thermal images illustrating surface and canopy temperature increases during EWC application. Warmer areas appear in orange/beige and cooler areas in purple/blue. These images provide visual context for heat generation during high‐voltage application, consistent with rapid tissue heating described for EWC.

Unlike other nonchemical approaches, EWC causes immediate and severe physiological injury: high‐voltage current rapidly heats intracellular water, leading to localized water vaporization, membrane rupture, protein denaturation and widespread cellular necrosis that extends into belowground tissues; high electric fields also may disrupt membrane integrity via electroporation, promoting ion leakage and irreversible cell damage.^23^, ^24^, ^25^, ^26^ Thermal images collected during field operation (Fig. 1) illustrate the rapid increase in aboveground tissue temperature during EWC, supporting the well‐documented mechanism of intracellular water vaporization and cellular rupture. Recent controlled‐environment research demonstrated that electrocution can raise internal plant temperatures >60–80 °C within seconds, with maximum temperature strongly predicting biomass and reproductive suppression across species and growth stages.^27^ Complementary glasshouse research showed that even low‐energy electrical pulses can induce progressive chlorosis, senescence, and mortality across multiple weed species.^28^

The present study evaluated a commercial electrical weed control (EWC) system, clopyralid, and integrated EWC‐clopyralid programs for *C. arvense* management in field conditions. We compared single *versus* sequential applications and assessed treatment effects on both aboveground shoot suppression and belowground root viability.

## 2 MATERIALS AND METHODS

### 2.1 Site description and experimental design

Four field studies were conducted at sites with naturally high densities of *C. arvense*. Two studies were conducted at the Oregon State University Lewis Brown Research Farm in Corvallis, Oregon (44.56° N, 123.22° W) in a fallow field previously used for blackberry (*Rubus* spp.) cultivation. Soil at the site was a Chehalis silty clay loam. Both studies were initiated in June 2023 in an area containing 52 shoots m^−2^ of *C. arvense*, with plants measuring ≈15–20 cm in height at the time of treatment. The infestation occurred as multiple dense patches within the field and study plots were established within the infested area to capture similar infestation levels among blocks.

Two other studies were conducted in June 2024 in a commercial hazelnut orchard in Canby, Oregon (45.18° N, 122.68° W) on Woodburn silt loam soil without irrigation. This field had a documented history of unsuccessful *C. arvense* management using clopyralid. The orchard was flail‐mown 3 weeks before treatment initiation. *C. arvense* plants were ≈5–7 cm tall at treatment with a density of 48 shoots m^−2^. These sites, in the Willamette Valley of Oregon, are characterized by mild, wet winters and warm, dry summers.

At each site, experiments were arranged as a randomized complete block design (RCBD) with four blocks, with treatments randomized within each block. Individual plots measured 3 × 6 m (18 m^2^). Plots were buffered by drive alleys to permit tractor operation and reduce interference among adjacent plots (≈3.7 m). In Corvallis, blocks were arranged linearly across the field; in Canby, plots were positioned adjacent to hazelnut rows within the infested orchard floor. Each site included two independent studies, conducted in adjacent fields with comparable infestation levels and *C. arvense* size. The studies were spatially separated by ≈50 m. Each study was individually randomized for treatment and independently conducted.

### 2.2 Treatment and equipment details

Treatments evaluated EWC and clopyralid either alone (single or sequential applications) or in combination, with EWC followed by clopyralid or clopyralid followed by EWC (Table 1). Clopyralid (Stinger®, Dow AgroSciences LLC, Zionsville, IN, USA) was applied either as a single application at 277 g a.i. ha^−1^ or as two sequential applications at 139 g a.i. ha^−1^ (totaling 277 g a.i. ha^−1^), as permitted by the label for use in hazelnut.^7^ The herbicide was applied using a CO_2_ pressurized backpack sprayer equipped with four air induction nozzles (AI‐11002; Teejet, Glendale Heights, KY, USA) and calibrated to deliver 187 L ha^−1^ at 275 kPa. The spray boom was positioned 50 cm above the weed canopy.

**Table 1:** Application rates, timing and dates of EWC and clopyralid treatments for *C. arvense* studies in Corvallis, OR and Canby, OR

Treatment	Application		Application dates			
	A (rate)	B (rate)	Corvallis I (App A/App B)	Corvallis II (App A/App B)	Canby I (App A/App B)	Canby II (App A/App B)
NTC^a^	‐	‐	‐	‐	‐	‐
EWC^b^	57 kJ m^2^	‐	14 June/‐	22 June/‐	7 June/‐	7 June/‐
Clop^c^	277 g ai ha^−1^	‐	14 June/‐	22 June/‐	7 June/‐	7 June/‐
EWC fb EWC	57 kJ m^2^	57 kJ m^2^	14 June/14 July	22 June/21 July	7 June/9 July	7 June/9 July
Clop fb Clop	139 g ai ha^−1^	139 g ai ha^−1^	14 June/14 July	22 June/21 July	7 June/9 July	7 June/9 July
EWC fb Clop	57 kJ m^2^	277 g ai ha^−1^	14 June/14 July	22 June/21 July	7 June/9 July	7 June/9 July
Clop fb EWC	277 g ai ha^−1^	57 kJ m^2^	14 June/14 July	22 June/21 July	7 June/9 July	7 June/9 July

^a^ NTC: nontreated control.

^b^ EWC: electrical weed control applied at 57 kJ m^2^ or 1 km h^−1^.

^c^ Clop: clopyralid applied at either 277 g ai ha^−1^ (single application) or two applications at 139 g ai ha^−1^. fb: followed in 4 weeks by another treatment application.

Electrical weed control was performed using a tractor‐mounted 30 kW commercial unit (EH‐30 Thor; Zasso, Zug, Switzerland) connected to a 77 kW tractor (5100GN; John Deere, Moline, IL, USA). Electricity was generated using a PTO‐driven 30 kW nominal‐power generator producing 220 V and 30 A of alternating current. The generator was connected to a three‐phase step‐up transformer. The transformer comprises three components: a primary winding, a secondary winding, and a magnetic core linking the windings. An AC voltage applied to the primary winding generates a time‐varying magnetic field in the core, which induces an electromotive force in the secondary winding according to Faraday's law of induction. The turns ratio between the windings determines the output voltage, allowing adjustment to match load requirements. The voltage output can be configured using two wiring methods and five voltage adjustment TAPs. The TAP is a physical lever that changes the effective number of turns in the primary winding, thereby altering the turns ratio. The delta wiring method delivers 3.9 to 7.6 kV across TAP settings (TAP 1 = 7.6 kV; TAP 5 = 3.9 kV), whereas the star winding method delivers 8.4 to 12 kV. Only the delta wiring configuration was used in this study because it allowed stable equipment performance under field soil conditions in Oregon.

Electric cables transmitted high‐voltage current to the front‐mounted applicator, which consisted of metal‐alloy electrodes (1.2 m wide) arranged in two rows spaced 0.5 m apart. The applicator included a pivoting arm that allowed electrodes to move around tree trunks. Electrodes remained in continuous contact with weed foliage and soil during operation (Fig. 1). The nominal electrical energy density per area applied is determined by the speed of operation using the following equation:

$$E\;\left(\mathrm{kJ}\;m^{-2}\right)=\text{Power}\;\left(\frac{\mathrm{kJ}}{s}\right)\times \mathrm{PF}\;X\frac{1}{\text{speed}\;\left(\frac{m}{s}\right)}\times \frac{1}{\text{width}\left(m\right)}$$

where E = energy (kJ m^−2^), Power = the generator power (kW = kJ s^−1^), PF = power factor, the ratio of real power (kW) to apparent power (kVA) in an AC system, speed = speed of operation (m s^−1^), and width = width of the treated area (m). The manufacturer‐specified PF for the generator–transformer system was used in all calculations.

All EWC applications were delivered at a standardized operational speed of 1 km h^−1^, corresponding to a nominal energy dose of 57 kJ m^−2^ or 570 MJ ha^−1^. Treatment descriptions and timings are shown in Table 1. Voltage selection was determined by soil moisture conditions, which limited the maximum voltage that could be applied before triggering equipment safety shutdowns. When soil electrical conductivity is high (wet soil conditions), the soil provides a low‐resistance pathway for the electrical current. Consequently, a substantial portion of the electrical energy is dissipated into the soil rather than transmitted through the plant. This low‐resistance pathway requires a rapid increase in current, causing a spike in the magnetic field of the equipment. If this surge is not interrupted, it can cause overheating of electrical components. Circuit breakers respond to these events by detecting the magnetic surge and opening the circuit to halt the flow of electricity, thereby initiating protective equipment shutdowns. In these studies, transformer voltage settings ranged from 4.7 to 5.6 kV.

### 2.3 Soil moisture threshold determination

In order to guide selection of voltage settings for field application and ensure stable operation of the electrical weeder (Zasso EH‐30), a soil‐moisture operating threshold was determined at the Lewis Brown Research Farm (Corvallis, OR, USA). This assessment was conducted in a separate area of the farm and was not part of the *C. arvense* field studies. The field section was irrigated for 12 h to establish a near‐saturated starting condition. Volumetric water content was measured in the upper 10 cm using a HydroSense II probe (Campbell Scientific, N Logan, UT, USA) immediately after irrigation and on subsequent days as the soil dried under ambient conditions and without further irrigation. The electrical weeder was then operated across areas with distinct moisture levels while voltage and amperage were monitored continuously. The maximum soil moisture permitting stable operation at each voltage tap was recorded and used to guide subsequent field applications.

### 2.4 Weed control and root fragment viability

*Cirsium arvense* control was evaluated at 14, 28 and 56 days after the initial treatment (DAIT) on a scale of 0–100% (0% = no control, 100% = complete control). Ratings were made at plot level within the treated strip. At 56 DAIT, aboveground biomass was collected from two 0.25‐m^2^ quadrats per plot in the Canby orchard. Shoots and capitula were separated, dried and weighed. Capitula biomass served as an indicator of reproductive output. Number of emerged shoots m^−2^ (density) also was recorded at each evaluation date. Biomass data were not collected at the Corvallis location due to site‐specific operational constraints.

At Corvallis, root fragments were collected at 56 DAIT from two separate field studies established in distinct areas of the field (Study 1 and Study 2). Within each plot, soil was excavated in areas where *C. arvense* shoots were present to recover root fragments. Then 10‐cm fragments were planted in glasshouse flats containing a peat‐based substrate (Sunshine #4; Sungro, Agawam, MA, USA). Root fragment length was standardized at 10 cm because available root material varied across plots, and longer fragments could not be collected consistently among treatments. Day/night temperatures were maintained at 24 °C and 15 °C. Each flat contained 10 fragments, and four replicate flats per treatment were established in each study (– = 8 flats per treatment across the two studies). Emergence (presence/absence of new shoots) was recorded for 4 weeks, and final shoot biomass was measured at the end of the study.

### 2.5 Statistical analysis

Statistical analyses were performed in R (v4.3.1).^29^ Site, treatment and DAIT were considered fixed effects, with DAIT treated as a categorical factor to account for repeated assessments over time. Experimental unit (plot for field experiments; flat for glasshouse experiments) and blocks were treated as random factors nested within an experimental run to account for variation among studies. When data were combined across the two studies within a site, this resulted in eight experimental units per treatment.

Individual percentage control scores were converted to proportions by dividing by 100. Because the beta distribution requires response values strictly between 0 and 1, observations equal to 1 were adjusted to 0.999 prior to analysis. Observations equal to zero were retained and modeled using a zero‐inflated beta distribution.^30^ Data were then analyzed using a generalized linear mixed model (GLMM) with a beta distribution in the glmmtMB package.^31^ Shoot density and root fragment emergence were analyzed using GLMMs with a negative binomial distribution, which were selected to accommodate overdispersed count data. Model assumptions were evaluated using simulated residuals with the DHARMa package.^32^ As biomass data were collected only at Canby, the model structure was modified to exclude ‘Site’ as a factor. These data were analyzed using a linear mixed‐effects model (*lmer* function, lme4 package)^33^ after applying a log transformation to meet normality and homoscedasticity assumptions.^34^ When treatment effects were significant (α = 0.05), Tukey's HSD‐adjusted pairwise comparisons were conducted using the emmeans package.^35^ Additionally, three sets of preplanned, orthogonal contrasts were evaluated: (i) single *versus* sequential EWC applications, (ii) EWC‐clopyralid *versus* clopyralid‐EWC, and (iii) sequential EWC and sequential clopyralid *versus* the combined‐program (EWC‐clopyralid and clopyralid‐EWC) treatments. The Bonferroni correction was applied to the preplanned contrasts to control the family‐wise Type I error rate given the limited number of comparisons.

## 3 RESULTS

### 3.1 Soil moisture threshold determination

Stable operation and the maximum achievable transformer voltage output were strongly influenced by soil moisture and TAP setting (Table 2). At higher soil moisture (27.6%), the equipment operated continuously only at TAP 5 (3.9 kV) and TAP 4 (4.7 kV). At this moisture level, TAP 3 could not sustain the flow of current. When soil moisture decreased to 25.7%, the equipment operated without interruption at 5.6 kV (TAP 3). Higher voltage settings (TAP 2 and TAP 1 > 7 kV) required drier soil (14.4%) for stable equipment operation.

**Table 2:** Maximum voltage output and the electrical weeder (Zasso EH‐30) TAP settings according to soil moisture levels conditions in silt loan soils in Oregon

TAP	Soil moisture (%)	Transfomer voltage (kV)	Generator amperage (A)
5	27.6	3.9	40
4	27.6	4.7	50
3	25.7	5.6	75
2	14.4	7.1	20 (60 max)
1	14.4	7.7	25 (90 max)

At high voltage levels, especially TAP 1 and 2, the electrical system was more sensitive to localized weed density. When the electrodes contacted dense patches of *C. arvense*, current increased sharply, reaching 60–90 A. The rise in current was accompanied by a drop in voltage and an audible change in the tractor's engine load, which is likely to have been caused by an increase in electrical demand on the generator during application. Higher weed density and aboveground biomass reduced electrical resistance by increasing conductive contact and creating additional pathways for the current to flow, resulting in current spikes and increased electrical demand on the generator.

In addition to subsurface moisture, surface soil condition influenced the distribution of alternating current in the soil profile. When the upper 2–3 cm of soil were visibly dry and powdery, the system operated with fewer protective shutdowns. Because dry soil has lower electrical conductivity than moist soil, a dry surface layer is likely to have reduced current leakage into the soil and increased the effective electrical resistance of the soil pathway, thereby shifting a greater proportion of current through plant tissue during electrode contact. The extent to which surface dryness alters current partitioning between plant and soil under field conditions warrants further study.

### 3.2 Weed control and root fragment viability

Given that the site × treatment × DAIT interaction was significant for control (*P* < 0.001) and the site × treatment interaction was significant for shoot density (*P* = 0.0037), results are presented separately by site. In Corvallis, EWC and clopyralid treatments ranged from 37% to 98% across evaluation dates, whereas the nontreated control remained at 0% (Table 3). At 14 DAIT, EWC‐based treatments provided 84–89% control, whereas clopyralid treatments provided low *C. arvense* control (37–53%), with the lowest control observed for the split‐rate, sequential application program (139 g a.i. ha^−1^ followed by 139 g a.i. ha^−1^; 37%). This is consistent with the slower symptom development expected for a systemic auxinic herbicide at early evaluation timing. *C. arvense* control increased among all treatments by 28 DAIT, ranging from 68% to 93%. EWC and clopyralid at 277 g a.i. ha^−1^ provided 82–93% control of *C. arvense*, whereas clopyralid at 139 g a.i. ha^−1^ was less effective, controlling 68% of *C. arvense*. At 56 DAIT, EWC applied alone provided relatively low *C. arvense* control (39% with a single application and 53% with sequential applications), whereas treatments that included clopyralid provided substantially higher control (≥95%), including clopyralid alone and integrated EWC–clopyralid programs (Table 3). Shoot density was reduced by 70–100% in treatments containing clopyralid, either alone or in combination with EWC, relative to the nontreated control, with clopyralid‐only treatments eliminating shoots at the final evaluation. In the combined treatments, regrowth of *C. arvense* shoots was observed after an initial application of EWC. However, the subsequent application of clopyralid improved control (89%). When clopyralid was applied first, control after the EWC was 98%. No flower production was observed in either study in Corvallis except for the nontreated control.

**Table 3:** Effect of electrical weed control (EWC) and clopyralid on *Cirsium arvense* control and shoot density in Corvallis, OR (2023) and Canby, OR (2024)^a^

	Corvallis				Canby			
Treatment	Control			Density	Control			Density
	14 DAIT^a^	28 DAIT	56 DAIT	56 DAIT	14 DAIT	28 DAIT	56 DAIT	56 DAIT
	(%)			(shoot m^−2^)	(%)			(shoot m^−2^)
NTC^b^	0d^†^	0e	0d	54a	0b	0b	0e	51a
EWC^c^	89a	93a	39c	33ab	93a	63a	38d	46a
Clop^d^ (277)	53b	82bc	96a	0d	92a	68a	43d	27ab
EWC fb EWC	87a	92a	53c	27ab	92a	66a	94b	26ab
Clop (139) fb Clop (139)	37c	68d	95ab	0d	90a	56a	78c	23ab
EWC fb Clop (277)	84a	90ab	89b	16b	94a	67a	87bc	13b
Clop (277) fb EWC	50bc	79cd	98a	2c	92a	58a	98a	11b

^a^DAIT: days after initial treatment. ^b^NTC: nontreated control. ^c^EWC: electrical weed control applied at 57 kJ m^2^ or 1 km h^−1^. ^d^Clop: clopyralid at 139 or 277 g ai ha^−1^. fb: followed by.

^†^ Means followed by the same letter within a column are not different based on Tukey HSD test (*α* = 0.05).

In Canby, all treatments provided excellent initial control of *C. arvense*, with control values exceeding 90% at 14 DAIT (Table 3). By 28 DAIT, control declined to 56–68% and treatment means did not differ. By 56 DAIT, the efficacy of single applications was reduced, falling below 43%, whereas sequential and integrated programs maintained higher control (78–98%). Among integrated programs, clopyralid followed by EWC provided 98% control compared with 87% for EWC followed by clopyralid, and this pattern was reflected in lower shoot density (11 *versus* 51 shoots m^−2^ for the nontreated control at 56 DAIT) (Table 3). Initial control with a single application of clopyralid at 277 g a.i. ha^−1^ was 92 and 68% at 14 and 28 DAIT, respectively; however, *C. arvense* regrowth was observed, and control was 43% at 56 DAIT. Overall, sequential treatments more effectively limited regrowth over time.

Biomass assessments at 56 DAIT indicated that shoot regrowth differed among programs (Table 4). Single treatments (EWC and clopyralid) and sequential clopyralid treatments (139 g a.i. ha^−1^) did not differ from the nontreated control. By contrast, repeated applications of EWC or integrated treatments lowered shoot biomass by 81–95%. For capitula biomass, single application of clopyralid, and all programs containing sequential and/or integrated applications reduced capitula compared to the nontreated control, including complete suppression (0 g m^−2^) in several treatments (Table 4).

**Table 4:** Shoot and capitula biomass of *Cirsium arvense* following electrical weed control (EWC) and clopyralid treatments collected 56 days after initial treatment in Canby, OR (*n* = 8)

Treatment	Shoot	Capitula
	(g m^−2^)	
NTC^a^	233 a^†^	10.6 a
EWC^b^	138 ab	5.2 ab
Clop^c^ (277)	122 ab	0.9 b
EWC fb EWC	11 c	0 c
Clop (139) fb Clop (139)	78 ab	0 c
EWC fb Clop (277)	44 bc	0 c
Clop (277) fb EWC	13 c	0.5 b

^a^ NTC: Nontreated control.

^b^ EWC: Electrical weed control applied at 57 kJ m^2^ or 1 km h^−1^.

^c^ Clop: clopyralid at 139 or 277 g ai ha^−1^. fb: followed by.

^†^ Means followed by the same letter within a column are not different based on Tukey HSD test (*α* = 0.05).

**Figure 2:**
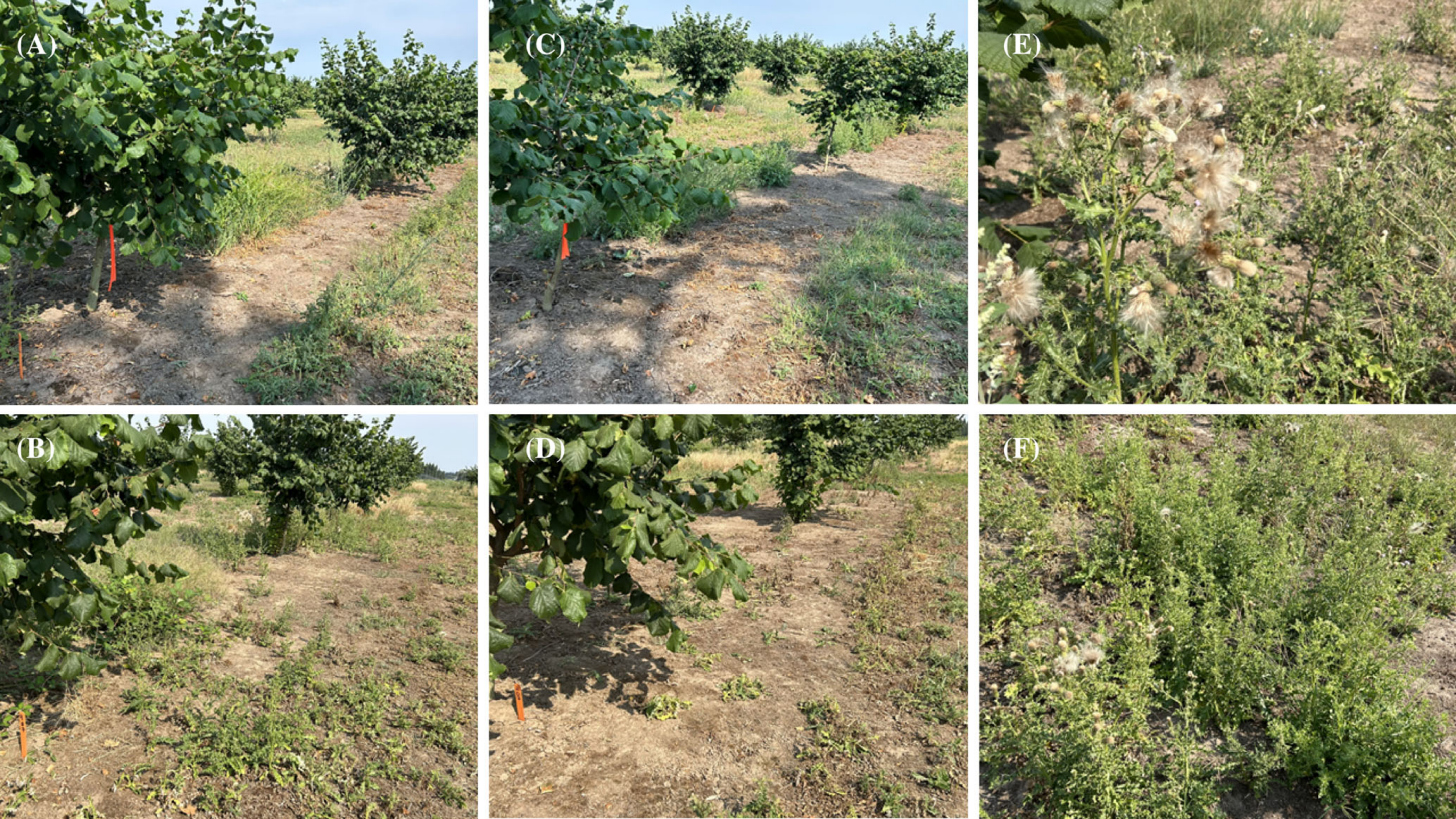
Field evaluation of *C. arvense* response to EWC and clopyralid treatments in a hazelnut orchard in Canby, OR (2024). (A) Sequential application of EWC (EWC 2×) showing substantial reduction in shoot regrowth. (B) Single application of EWC (EWC 1×) with noticeable shoot regrowth. (C) Clopyralid (277 g a.i. ha^−1^) followed by EWC (Clop fb EWC), exhibiting effective shoot suppression. (D) EWC followed by clopyralid (EWC fb Clop). (E–F) Nontreated control with *C. arvense* flowering and seed production and high density of shoots.

Glasshouse evaluations of root fragments corroborated field observations (Table 5). All treatments (EWC, clopyralid and their combinations) were equally effective, reducing new shoot emergence by 71–100% and shoot dry weight (DW) by 76–94% relative to the nontreated control. This demonstrates that EWC affected *C. arvense* roots in a similar way to clopyralid. Root bud control is a key component for sustained control of this species, depleting *C. arvense* root reserves. Contrast analyses confirm that two applications of EWC significantly improved *C. arvense* control compared to a single application, reducing the odds of survival by 84% [odds ratio (OR) = 0.16, *P* < 0.001] (Table 6). Sequential EWC applications reduced *C. arvense* shoot biomass by 12‐fold compared to a single pass (*P* < 0.001). These differences in shoot suppression between single and sequential EWC applications are depicted in Fig. 2(A), (B). A comparison of combined treatments indicated that clopyralid followed by EWC provided greater control compared to EWC followed by clopyralid (OR = 0.16, *P* < 0.001), and shoot density was markedly lower when clopyralid preceded EWC [*P* < 0.001; Fig. 2(C), (D)]. Finally, combining EWC and clopyralid achieved significantly higher control (87–98%) than either method applied twice (53–94% for EWC, 78–95% for clopyralid; *P* < 0.001).

**Table 5:** Emergence and dry weight of *Cirsium arvense* shoots in the greenhouse from field‐collected root fragments following electrical weed control (EWC) or clopyralid treatment in Corvallis, OR (*n* = 8). Root fragments were collected 56 days after initial treatment

Treatment	New shoot emergence	Weight of emerged shoots
	(%)	(g)
NTC^a^	85 a^†^	56 a
EWC^b^	15 b	4.8 b
Clop^c^ (277)	8 b	4.2 b
EWC fb EWC	25 b	13.2 b
Clop (139) fb Clop (139)	0 c	0 c
EWC fb Clop (277)	4 b	0 c
Clop (277) fb EWC	5 b	4.1 b

^a^ NTC: Nontreated control.

^b^ EWC: Electrical weed control applied at 57 kJ m^2^ or 1 km h^−1^.

^c^ Clop: Clopyralid at 139 or 277 g ai ha^−1^. fb: followed by.

^†^ Means followed by the same letter within a column are not different based on Tukey HSD test (*α* = 0.05).

**Table 6:** Contrast analysis between selected electrical weed control treatments on *Cirsium arvense* control at 56 days after treatment, shoot density, biomass, and root fragment emergence in Corvallis and Canby, OR

Contrast	Response	Odds ratio	SE	Test statistic	*p*‐value
EWC^a^ 1× *vs* 2×	Control	0.16	0.03	−8.03	**<0.001**
	Shoot density	1.68	0.55	1.59	0.111
	Biomass^†^	12.11	4.84	6.22	**<0.001**
	Root fragment emergence^‡^	0.58	0.22	−1.41	0.157
EWC fb Clop^b^ *vs* Clop fb EWC	Control	0.14	0.04	−6.30	**<0.001**
	Shoot density	3.49	1.29	3.39	**<0.001**
	Biomass^†^	3.37	1.51	2.70	**0.010**
	Root fragment emergence^‡^	0.77	0.77	−0.32	0.748
EWC and Clop 2× *vs* Integrated method	Control	0.30	0.06	−5.81	**<0.001**
	Shoot density	1.21	0.30	0.76	0.449
	Biomass^†^	1.23	1.23	0.69	0.493
	Root fragment emergence^‡^	<0.01	<0.01	0.00	1.00

^a^ EWC: electrical weed control applied at 57 kJ m^2^ or 1 km h^−1^ once or twice.

^b^ Clop: clopyralid (277 g ai ha^−1^). fb: followed by. Ratio: odds ratio for control, shoot density and root fragment emergence and response ratio for biomass; values >1 indicate higher odds or response in the first treatment. Test statistic = *z*‐value for GLMMs and *t*‐ratio for LMMs. SE = standard error. Significant contrasts are in bold.

^†^ Biomass data from Canby studies only (*n* = 8).

^‡^ Emergence of new shoots from root fragments based on Corvallis studies only (*n* = 8).

## 4 DISCUSSION

To the best of our knowledge, this is the first published field study evaluating EWC for the management of *C. arvense* and comparing its performance with clopyralid‐based programs. Clopyralid remains one of the most effective herbicides for the management of *C. arvense*,^36^, ^37^, ^38^ and our results align with previous findings demonstrating strong reductions in shoot density, seed production and belowground regrowth potential.^5^, ^39^ In our study, clopyralid‐based programs generally outperformed EWC at Corvallis, whereas at Canby, sequential EWC applications provided short‐term control comparable to a single clopyralid treatment. These site‐specific differences reflect the biological and environmental complexity of perennial weed management.

Electrical weed control alone reduced aboveground biomass, capitula production, and root fragment viability of *C. arvense* when applied twice, although EWC‐only programs were less consistent than programs that included clopyralid at 56 DAIT. Across sites, two EWC passes resulted in 53% control in Corvallis and 94% control in Canby at 56 DAIT, consistent with the preplanned contrast showing improved control with sequential *versus* single EWC applications. The challenges of targeting *C. arvense* roots are well‐documented, as effective long‐term suppression requires injury to numerous adventitious buds distributed laterally and vertically within the soil profile.^40^, ^41^ Evaluating control at 120–180 DAIT was not feasible in this study because plants enter natural dormancy by early fall in western Oregon, and the absence of new shoot emergence after this point reflects seasonal phenology rather than treatment effects. Our single‐season results demonstrate short‐term injury to these regenerative structures, concentrated in the upper 60 cm of soil,^42^ but multiseason evaluations will be required to determine the degree to which EWC can contribute to long‐term population decline.

The difference in treatment performance between sites is likely to have been influenced by a combination of plant size and stand at application. Although initial *C. arvense* shoot densities were similar at Corvallis and Canby, plants were smaller at Canby at the time of treatment because the orchard floor had been flail‐mown before study initiation as part of the farm standard management. Reduced canopy size is likely to have increased effective electrical current density within remaining plant tissues, thereby increasing electrical injury and plant mortality, helping explain why EWC was more effective at Canby than at Corvallis at 56 DAIT.

The greatest control in our field trials was observed in integrated EWC‐clopyralid programs, and treatment sequence influenced performance. This sequence reduced shoot densities more effectively than EWC alone or clopyralid alone at both sites, although clopyralid followed by clopyralid also performed well at the Corvallis site. Clopyralid is likely to have thinned the stand and weakened surviving stems before the subsequent electrical treatment. At the time of EWC application in clopyralid‐treated plots, many shoots were wilted and partially collapsed, which is likely to have altered electrode–plant contact and reduced simultaneous contact of multiple shoots compared with dense, upright stands in nontreated control. In nontreated control plots, the first EWC pass encountered dense vegetation where many stems contacted the electrodes at once. This is consistent with electrical current partitioning in heterogeneous plant–soil matrices, whereby each stem–root–soil column acts as a parallel conductive pathway, so dense vegetation reduces the equivalent resistance of the plant–soil network and increases total current draw but subdivides current among many stems, lowering current density and tissue heating in individual plants.^23^, ^24^, ^27^, ^28^ Such patterns illustrate how treatment sequence influences electrical load and therefore EWC performance.

While no published studies have specifically examined EWC efficacy on *C. arvense*, results from other cropping systems help interpret our findings. A study in vineyards evaluated EWC using a direct‐current unit delivering up to 36 kW (Xpower; Zasso) concluded that EWC performed comparably to herbicide applications and more effectively than mowing across mixed weed communities.^22^ Likewise, in organic blueberry systems, species‐specific evaluations showed that EWC (Xpower and Raiden; Zasso) achieved 85–100% control of upright broadleaf species such as *Epilobium ciliatum* and *Persicaria pensylvanica*, whereas grasses and prostrate broadleaf weeds (e.g. *Festuca arundinacea* and *Kickxia elatine*) required multiple applications and higher energy doses to achieve moderate control (67–73%).^43^ These patterns are consistent with our observation of strong early injury following EWC but reduced durability when EWC was used alone at 56 DAIT in Corvallis, which is likely to reflect regrowth from extensive belowground buds and reserves characteristic of perennial weeds.^38^, ^40^, ^41^

In annual cropping systems, EWC has been evaluated using tractor‐mounted equipment such as the Weed Zapper 6R30 unit (Old School Manufacturing LLC, Sedalia, MO, USA). This system delivers up to 110 kW of alternating current through a 3‐m horizontal electrode positioned above the crop canopy and operating at speeds between 3.2 and 6.4 km h^−1^, resulting in estimated energy doses of 5.8–24.7 kJ m^−2^.^44^ In one study, EWC efficacy at 42 days after treatment ranged from 51–93% for *Amaranthus tuberculatus*, 80–95% for *Ambrosia artemisiifolia* L., 39% for *Setaria pumila Poir*. and 54–57% for *Echinochloa crus‐galli* (L.) P. Beauv.^44^ Weed control varied among species and was primarily influenced by plant size and moisture content at the time of treatment. A similar study using comparable equipment reported ≤87% control of *Amaranthus palmeri*, with two EWC passes generally providing greater control than a single application.^45^ This aligns with our planned contrasts showing improved outcomes with sequential EWC applications and supports the conclusion that treatment repetition and stand structure at application strongly influence EWC effectiveness in *C. arvense*.

By contrast, perennial weeds tend to be less affected by EWC. In field trials on yellow nutsedge (*Cyperus esculentus* L.) in Belgium, a single application using a Zasso XP 300 tractor‐mounted unit operating at 7 kV and delivering 2 kW m^2^, and Rootwave Pro unit operating at 5 kV (~2 s treatment) reduced aboveground shoot vitality by up to 91–100% and aboveground shoot control at speeds of 1.1–1.5 km h^−1^ (corresponding to 4.8–6.5 kJ m^–2^). Nevertheless, subterranean tubers remained unaffected by a single treatment, highlighting the challenge of targeting subterranean structures in perennial weeds.^46^ Complementary research in the Pacific Northwest using tractor‐powered EWC systems (alternating‐ and direct‐current) applied at 24 to 518 kJ m^−2^ (operational speeds of 0.5–3.0 km h^−1^) showed that although single applications often allowed regrowth, sequential EWC or mowing followed by EWC improved sustained control (80–93% by 80 DAIT) and also reduced tuber emergence, tuber density and weight relative to the nontreated control, emphasizing that treatment repetition and treatment order are critical for control of perennial weeds with subterranean propagules.^47^

The role of subterranean structures in increasing plant tolerance to EWC was noticeable in a recent study under controlled benchtop conditions considering *Sorghum halepense* control by an EWC system.^48^ Plants propagated from both seeds and rhizomes were treated once with increasing doses of direct current at the 2‐, 4‐ and 6‐leaf stages. *S. halepense* at the first true leaf stage required only 0.35 kJ plant^−1^ for 90% biomass reduction but required increasingly greater energy doses as maturity advanced. The energy required to achieve the same effect increased by 10.7‐ to 31.4‐fold at the 4‐ and 6‐leaf stages, respectively.

Despite the improved efficacy of clopyralid and EWC, this approach is unsuited to organic production. Replacing clopyralid with organic herbicides may not yield comparable results because the latter are not systemic, and in general are not cost‐effective options in organic perennial crops.^49^ Other nonchemical methods may be suitable for combination with EWC. A common practice for managing *C. arvense* in some annual and organic cropping systems is repeated tillage, typically deep and closely timed cultivations performed several times during the growing season, although this approach is not suitable for orchards.^50^ Repeated mowing combined with a smothering summer cover crop can reduce *C. arvense* shoot density^51^ Effective suppression requires cover crop species that grow vigorously in summer, produce tall canopies, and cover almost all of the soil surface to limit light availability and to strongly compete for water.^52^ However, this approach is not suitable for tree nut orchards because summer cover crops typically inhibit harvest operations, as orchard floors must remain clean and free of dense vegetation during late summer.^53^ The more compatible winter cover crops do not provide the same level of summer competition needed to suppress *C. arvense*.

Combining mowing with EWC may offer an optimal strategy for orchard systems. Mowing is a widely adopted method for weed management and can be implemented throughout much of the growing season.^54^ When combined with EWC, this approach may be more energy‐efficient than either method alone. For example, flail mowing has an estimated energy demand of 3.1–5.7 kJ m^2^ per pass at a speed of 7 km h^−1^.^55^, ^56^ Although effective in reducing weed biomass and seed production, mowing typically requires five to seven passes for adequate suppression and is less effective against prostrate weed species.^45^, ^57^ By contrast, EWC is a more energy‐demanding method, requiring ≈54 kJ m^2^ at 1 km h^−1^ and treating only a 1.2‐m swath per pass.^58^ However, it provides excellent control across multiple species. Although not tested in this study, this hypothesis aligns with our field observations and is supported by previous reports, which have demonstrated that pre‐treatment with mowing increased EWC efficacy by reducing canopy volume and improving contact between electrodes and remaining stems.^47^

Our current understanding of the long‐term effects of *C. arvense* control strategies is limited. Therefore, multiyear studies are needed to compare the impacts of different methods on weed population dynamics and energy requirements.^59^ We propose that energy‐use comparisons should be standardized as energy per unit area per year (e.g. MJ ha^−1^ year^−1^), because the energy required to maintain effective control is likely to decrease over time as weed density declines.

A cost comparison of treatments was beyond the scope of this study owing to its limited duration. Currently, there are no detailed studies evaluating the operational costs of EWC in orchard systems. However, the narrow treatment width (1.2 m) of current EWC equipment increases the number of passes needed to cover a typical tree nut orchard. While scaling up equipment size could improve efficiency, this approach may be constrained by tractor power limitations. The largest tractors compatible with orchard layouts typically provide up to 89.5 kW (120 hp), and higher‐capacity equipment incurs greater acquisition and operating costs. The average Oregon hazelnut farm is ≈40 ha in size and typically operates tractors rated ≈55 kW (75 hp).^60^ An alternative approach could be to use lower‐power EWC units with narrower widths or slower travel speeds, designed specifically for standard orchard tractors; although this would increase treatment time, it might reduce capital outlay and make EWC more accessible to smaller farms.

## 5 CONCLUSION

This study is the first to quantify the effects of EWC on *C. arvense* in orchard systems. Across two locations in Oregon, EWC reduced shoot density and decreased root bud viability, with the most consistent reductions occurring when EWC was applied twice. Although EWC did not always match the performance of clopyralid, particularly at the Corvallis site, sequential EWC applications provided levels of suppression comparable to a single clopyralid application in Canby. These results demonstrate that EWC can disrupt aboveground and belowground regenerative structures and, when used strategically, may contribute to integrated management programs for perennial weeds in orchard systems.

The performance of EWC was influenced by the number and sequence of applications, with the most effective outcomes observed in sequential and integrated treatments programs. Integrating EWC with clopyralid provided greater control than two applications of either method alone, underscoring the efficacy of combining methods within an integrated weed management program. Although this single‐season study demonstrated that EWC, especially when combined with clopyralid, can effectively control *C. arvense*, multiyear evaluations are needed to assess the durability of these effects. Additional research also is needed to identify nonchemical strategies that could be combined with EWC to improve operational feasibility and broaden its applicability across cropping systems.

## CONFLICT OF INTEREST

The authors declare no conflict of interest.

## Data Availability

The data that support the findings of this study are available from the corresponding author upon reasonable request.
